# Multimodal Neuroimaging Study of Visual Plasticity in Schizophrenia

**DOI:** 10.3389/fpsyt.2021.644271

**Published:** 2021-04-01

**Authors:** S. Andrea Wijtenburg, Jeffrey West, Stephanie A. Korenic, Franchesca Kuhney, Frank E. Gaston, Hongji Chen, Laura M. Rowland

**Affiliations:** Maryland Psychiatric Research Center, Department of Psychiatry, University of Maryland School of Medicine, Baltimore, MD, United States

**Keywords:** visual plasticity, schizophrenia, fMRI, GABA, glutamate, healthy adults, magnetic resonance spectroscopy

## Abstract

Schizophrenia is a severe mental illness with visual learning and memory deficits, and reduced long term potentiation (LTP) may underlie these impairments. Recent human fMRI and EEG studies have assessed visual plasticity that was induced with high frequency visual stimulation, which is thought to mimic an LTP-like phenomenon. This study investigated the differences in visual plasticity in participants with schizophrenia and healthy controls. An fMRI visual plasticity paradigm was implemented, and proton magnetic resonance spectroscopy data were acquired to determine whether baseline resting levels of glutamatergic and GABA metabolites were related to visual plasticity response. Adults with schizophrenia did not demonstrate visual plasticity after family-wise error correction; whereas, the healthy control group did. There was a significant regional difference in visual plasticity in the left visual cortical area V2 when assessing group differences, and baseline GABA levels were associated with this specific ROI in the SZ group only. Overall, this study suggests that visual plasticity is altered in schizophrenia and related to basal GABA levels.

## Introduction

Schizophrenia (SZ) is a severe psychiatric illness with well-documented visual learning and processing deficits (1–3), which may be due to alterations in long-term potentiation (LTP). LTP is a basic cellular plasticity mechanism underlying learning and memory, and it has been previously studied in a variety of regions including the hippocampus and visual cortex using high frequency electrical or visual stimulation, respectively (4–7). Clapp et al. adapted a visual plasticity paradigm that employed low and high frequency stimulation known to induce LTP-like changes in rodents and successfully tested the paradigm in healthy adults using EEG and fMRI. In these studies, increased visual evoked potentials (8) or increased fMRI BOLD activation (9) were observed following high frequency stimulation, which was similar to previous animal studies. A similar visual plasticity paradigm has been used in several other studies in healthy adults and shown elevations in visual evoked potentials or fMRI BOLD activation post-high frequency stimulation (10, 11) as well as a variable response in fMRI BOLD activation post-high frequency stimulation (12).

Several studies have utilized visual paradigms to assess visual plasticity in adults with SZ vs. healthy controls using EEG. Cavus et al. demonstrated impaired visual cortical plasticity in adults with SZ compared to healthy controls as evidenced by a lack of persistent visual evoked potentials in the visual cortex post-high frequency stimulation (13). In a study where participants were given D-cycloserine or placebo, exploratory analyses by Forsyth et al. (14) showed impaired visual evoked potentials post-high frequency stimulation in adults with SZ who received placebo compared to healthy controls who received placebo. The visual plasticity response in the SZ group did not change with administration of 100 mg of D-cycloserine. D'Souza et al. reported an LTP-like enhancement with a dose of a glycine transporter-1 (~75% occupancy) in adults with SZ (15). Another study by Wynn et al. demonstrated a plasticity effect (visual evoked potential post-high frequency stimulation) in both healthy controls and adults with SZ; however, the groups were not significantly different (16). In other visual paradigms that employed monocular deprivation or sensory adaption, adults with SZ have significantly reduced visual evoked potential amplitudes compared to controls (17, 18). Thus, in the literature to date, there appears to be mixed results regarding whether visual plasticity is impaired in SZ.

Glutamate, the primary excitatory neurotransmitter in the human brain, is significantly involved in LTP such that it modulates NMDA receptors, which triggers a cascade of events leading to a long-lasting increase in signal transmission between neurons (4, 19). Currently, in humans, the only non-invasive methodology to quantify glutamatergic metabolites *in vivo* is proton magnetic resonance spectroscopy. This technique has been used to show altered levels of glutamatergic metabolites in several different brain regions in adults with SZ (20). In addition to glutamate, magnetic resonance spectroscopy can be used to measure glutamine, of which 80% is derived from glutamate involved in neurotransmission, and GABA, the primary inhibitory transmitter in the human brain and modulator of LTP *in vivo* (21). Gaining a better understanding of basal glutamate, glutamine, and GABA levels and the relationship to visual plasticity may provide insight into the mixed visual plasticity results in SZ and in the future, serve as a potential pharmacological treatment target.

The first aim of this study was to test the hypothesis that visual plasticity is reduced in SZ using fMRI. A second aim was to use a multimodal approach to determine whether baseline resting levels of occipital cortex glutamatergic metabolites and GABA as measured using magnetic resonance spectroscopy were related to visual plasticity response as assessed using fMRI. Given that glutamatergic function is altered in SZ and a previous study by our group in healthy controls showed that glutamine was related to visual plasticity (11), we hypothesized that the relationship between glutamine to visual plasticity would be weaker in adults with SZ compared to healthy controls. We expected the magnitude of the relationship between glutamate and visual plasticity as well as GABA and visual plasticity to be smaller in adults with SZ compared to healthy controls.

## Materials and Methods

All research was conducted at the University of Maryland Center for Brain Imaging Research (CBIR) at the Maryland Psychiatric Research Center. This study was approved by the University of Maryland Baltimore Institutional Review Board, and all participants (both SZ and healthy controls) provided written, informed consent prior to study initiation. Adults with SZ were evaluated for capacity to consent to ensure that each participant fully understood the study procedures. The healthy control data was previously reported in (11).

### Participant Characteristics

Seventeen adults with SZ and 18 healthy controls participated in the study. Participant demographics are described in Table 1. Adults with SZ were characterized and evaluated with the Structured Clinical Interview for DSM-IV (SCID), the Brief Psychiatric Rating Scale (BPRS) (22), and the Brief Negative Symptom Scale (BNSS) (23). For both groups, inclusion criteria for the study were: (1) no contraindication for magnetic resonance imaging scanning, (2) no current or past neurological condition, head trauma, or focal findings on an magnetic resonance imaging, or (3) no substance abuse in the past 6 months or lifetime dependence excluding nicotine. The healthy control group had no current or past psychiatric, neurological, or major medical disorders or substance abuse/dependence. Functional capacity was assessed using the UCSD Performance Based Skills Assessment (UPSA-2) (24). From the MATRICS Consensus Cognitive Battery (MCCB), verbal and visuo-spatial learning were assessed using the Hopkins Verbal Learning Task—Revised (HVLT) and the Brief Visuospatial Memory Task—Revised (BVMT) (25, 26).

**Table 1:** Participant characteristics, mean (standard deviation).

	**Schizophrenia**	**Healthy controls**	***p*-value**
Gender (M/F)	10/7	9/9	0.6
Age (years)	41.5 (15)	36.2 (16)	0.32
Education (years)	13.7 (2.3)	14.6 (1.6)	0.22
Illness duration (years)	20.4 (14)	N/A	
Smoker (Yes/No)	3/14	2/16	0.58
**Psychiatric ratings**			
BPRS (total)	39.7 (15.0)	N/A	
BPRS (positive)	8.9 (6.0)	N/A	
BPRS (negative)	7.1 (2.0)	N/A	
BNSS	17.5 (10.1)	N/A	
**Antipsychotics'**			
1st Generation	1	N/A	
2nd Generation	11	N/A	
Both	3	N/A	
None	2	N/A	
**Cognitive measures**			
HVLT	22.1 (6.3)	26.8 (3.6)	0.01^*^
BVMT	17.1 (9.1)	25.6 (5.1)	0.02^*^
UPSA	92.0 (11.5)	100.8 (9.6)	0.023^*^

* *p < 0.05, '– number of participants taking these medications, BNSS, Brief Negative Symptom Scale; BPRS, Brief Psychiatric Rating Scale; BVMT, Brief Visuospatial Memory Task; HVLT, Hopkins Verbal Learning Task; UPSA, UCSD Performance Based Skills Assessment*.

### Neuroimaging

A Siemens TIM Trio 3T MR system with a 32-channel phased array head coil was utilized for this study. The imaging protocol consisted of axial T_1_-weighted MP-RAGE images used for both magnetic resonance spectroscopy voxel and echo planar imaging placement, spectroscopy data acquisition, and finally fMRI.

**Figure 1:**
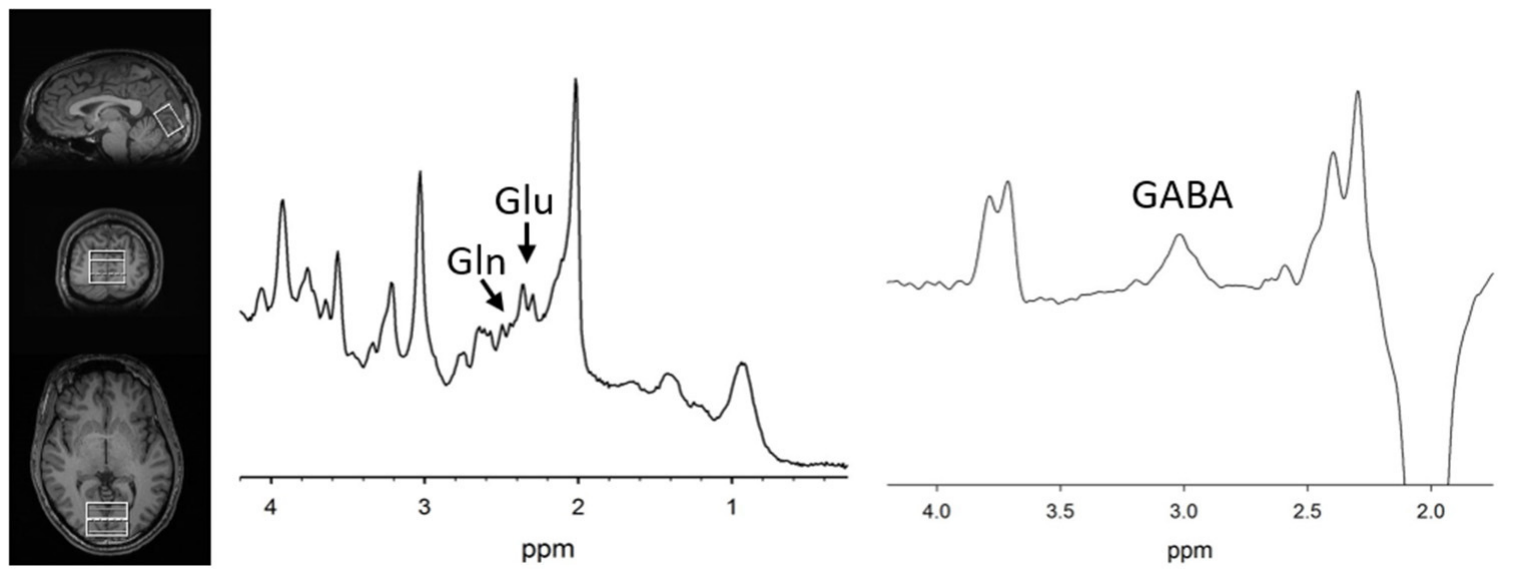
(Left) T1-weighted images showing voxel placement along the midline in the occipital cortex. Representative PR-STEAM spectrum (center) and MEGA-PRESS spectrum (right) from the same participant showing excellent quality data. tCr, total creatine; Glu, glutamate; Gln, glutamine; GABA, γ-aminobutyric acid.

MRS methods were previously described (11). Briefly, the magnetic resonance spectroscopy voxel was placed along the midline in the occipital cortex (Figure 1) in both groups. Participants were asked to rest but remain awake. To detect glutamate and glutamine, spectra were acquired with phase rotation STEAM (PR-STEAM) (27–30): TR/TM/TE = 2,000/10/6.5-ms, VOI ~ 3.0 × 4.0 × 2.0-cm^3^, NEX = 128, and water reference NEX = 16. Data were post-processed offline using in-house MATLAB code and then quantified in LCModel (6.3-0I). For GABA detection, a macromolecule–suppressed MEGA-PRESS sequence was utilized (31): TR/TE = 2,000/68 ms, VOI ~ 3.0 × 4.0 × 2.0-cm^3^, NEX = 256 (128 ON and 128 OFF), and water reference NEX = 16, and data were quantified in Gannet 2.0. In-house MATLAB code based on Gasparovic et al. (32) was used to calculate metabolite levels using water as a reference as well as correct for the proportion of the gray matter (GM), white matter (WM), and cerebrospinal fluid (CSF) within each spectroscopic voxel and relaxation effects. More details regarding quantification are outlined here (33). All metabolite levels are reported in institutional units. See Figure 1 for representative voxel placement and spectra.

fMRI methods were previously described (11). In brief, E-Prime 2.0 (Psychology Software Tools, Inc., Sharpsburg, PA, USA) was used to display the visual stimulus: a centrally located flashing checkerboard, based on based on previous studies (5, 8, 10, 12, 13) and similar to Cavus et al. (13) and Lahr et al. (12). The task involved two low frequency (0.9 Hz) stimulation runs, one high frequency (9 Hz) stimulation to induce visual plasticity, 2 min of rest, and two runs of low frequency stimulation. During the low frequency stimulation and high frequency stimulation, participants were asked to fixate on a centrally located crosshair except during the 2-min rest period where eyes were closed. During the low frequency stimulation blocks, echo-planar imaging data were acquired TR = 2,100 ms, TE = 27 ms, FOV = 220 × 220 mm, matrix size = 128 × 128, Number of slices = 39,143 measurements, voxel size = 1.7 × 1.7 × 4.0 mm). Echo planar imaging data were analyzed using MATLAB (R2013a) (The Mathworks Inc., Natick, Massachusetts) and Statistical Parametric Mapping (SPM) 8.0 (Wellcome Trust Center for Neuroimaging, Department of Cognitive Neurology, Institute of Neurology, London: http://www.fil.ion.ucl.ac.uk/spm).

First and second level models were previously described (11). Briefly, first level models were performed for each group, and second level modeling involved the following contrasts: (post-high frequency stimulation “on”—“off”) minus (pre-high frequency stimulation “on”—“off”). Second level analysis for both healthy controls and adults with SZ tested for visual plasticity *via* a one sample *t*-test, appropriate for within subject designs. A mask of the spectroscopic voxel was applied to restrict the analyses to only the magnetic resonance spectroscopy region.

### Statistical Analyses

Using SPSS 23.0 (IBM SPSS Statistics, Armonk, NY, USA), group differences were assessed among the demographic variables *via* chi-square or *t*-test as appropriate with significance set to *p* < 0.05. Normality assessments conducted *via* the Shapiro-Wilk-test revealed that the data were normally distributed. For the three metabolites of interest, *t*-tests with significance set at *p* < 0.05 were performed to examine differences between the two groups. Similarly, *t*-tests with significance set at *p* < 0.05 were also computed for magnetic resonance spectroscopy quality factors such as linewidth (LW), signal-to-noise ratio (SNR), and metabolite Cramer Rao Lower Bounds (CRLBs) or GABA fit errors to examine whether data were of similar quality between groups.

Visual plasticity within group differences were considered significant with a threshold set to *p* < 0.05 FWE-corrected. For between group visual plasticity differences, small volume correction with a region of interest diameter of 4 mm was applied to examine differences in visual plasticity between adults with SZ and healthy controls with significance threshold set to *p* < 0.05, Family Wise Error (FWE)-corrected.

To examine the relationship between visual plasticity and metabolites, correlations were computed using SPSS 27.0 (IBM SPSS Statistics, Armonk, NY, USA) between region of interest values extracted from significant activations using MarsBar (5) with 4 mm radius and the three metabolite levels (*p* = 0.05/3). In the SZ group, exploratory analyses were conducted to determine whether cognition function or symptom ratings related to visual plasticity.

## Results

### Demographics

The two groups were well-matched in terms of gender, age, education, and smoking (*p*'s > 0.05, Table 1). There were significant differences in terms of Hopkins Verbal Learning Test (*p* = 0.01), Brief Visuospatial Memory Test (*p* = 0.02), and UPSA-2 (*p* = 0.023) such that the SZ group performed worse on measures of memory and functional capacity compared to the healthy control group.

### MRS

**Figure 2:**
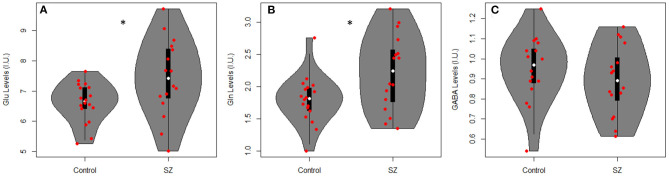
Violin plots with individual data points overlayed in red showing metabolite levels in institutional units (I.U.) for **(A)** glutamate (Glu), **(B)** glutamine (Gln), and **(C)** GABA in adults with SZ and healthy controls (Controls). There were significant differences between groups for Glu (*p* = 0.021) and Gln levels (*p* = 0.016) such that Glu and Gln were higher in SZ vs. healthy controls. *p < 0.05.

Table 2 summarizes mean metabolite levels and quality factors. One PR-STEAM dataset was not acquired, and three GABA datasets were excluded due to fit error > 15%. All glutamate and glutamine CRLBs from both healthy controls and SZ were <20 or 30%, respectively. Overall, data were of excellent quality in both groups, and there were no significant differences between groups for linewidth, signal-to-noise ratio, glutamate CRLB, glutamine CRLB, or GABA fit error (*p*'s > 0.05). There were significant differences between healthy controls and SZ for glutamate (*p* = 0.021) and glutamine levels (*p* = 0.016) such that glutamate and glutamine levels were higher in SZ compared to healthy controls as shown in Figure 2. There were no differences in GABA levels between groups (*p* = 0.22).

**Table 2:** Magnetic resonance spectroscopy metabolite means (standard deviation).

**Metabolites (I.U.)**	**Schizophrenia**	**Healthy controls**	***p*-value**
Glutamate (Glu)	7.4 (1.3)	6.6 (0.7)	0.021^*^
Glutamine (Gln)	2.2 (0.6)	1.8 (0.4)	0.016^*^
GABA	0.87 (0.2)	0.95 (0.2)	0.22
Linewidth (LW)^a^	0.046 (0.02)	0.042 (0.01)	0.55
Signal-to-Noise Ratio (SNR)^a^	73 (18)	76 (16)	0.67

* *p < 0.05, 1 Glu and 1 Gln were excluded, 3 GABA datasets were excluded*.

a *LW and SNR as computed by LCModel*.

*I.U., institutional units*.

### fMRI

For the healthy control group, visual plasticity was observed in dorsal V2 and V3 and positively related to glutamine with details reported elsewhere (11). There were no significant regions of interest showing visual plasticity in the SZ group that survived FWE-correction. The opposite contrast of pre-high frequency stimulation vs. post-high frequency stimulation yielded no significant regions in both healthy controls and SZ.

**Figure 3:**
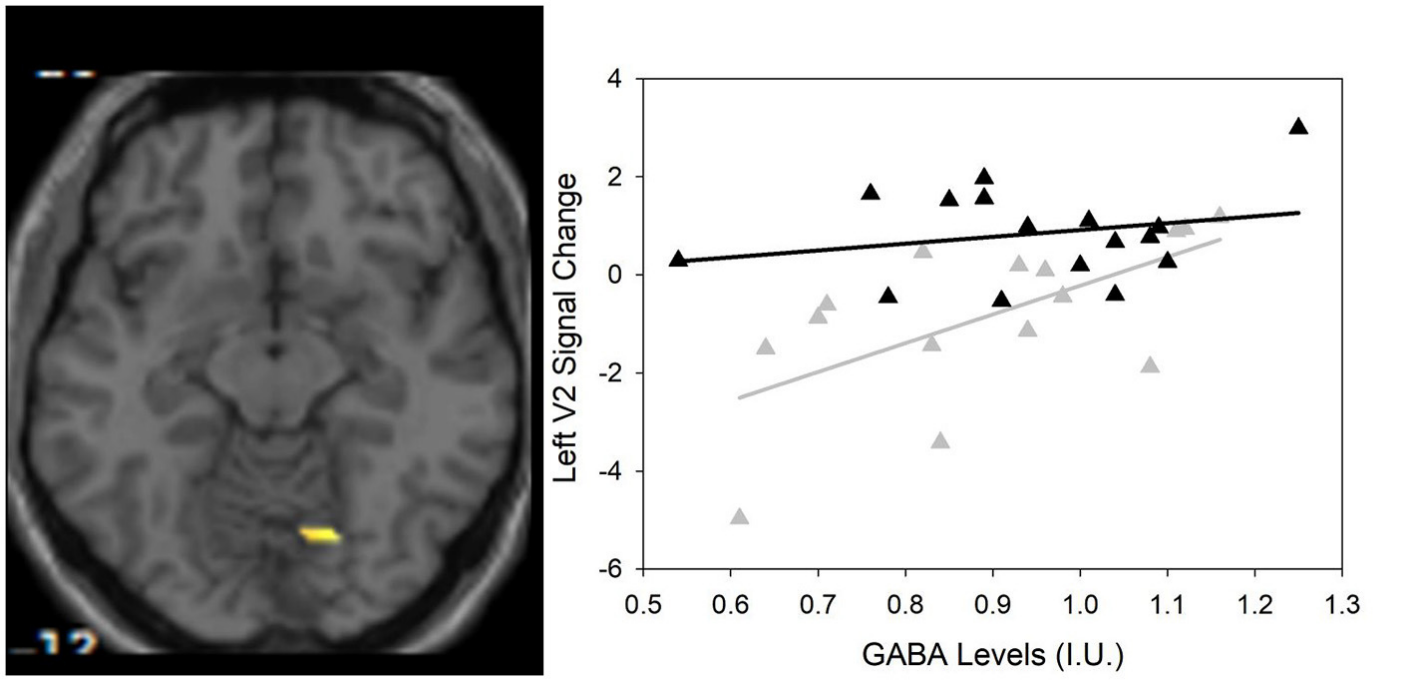
(Left) Group differences in visual activation within the magnetic resonance spectroscopy voxel between healthy controls and SZ using small volume correction that survived Family Wise Error (FWE)-correction (*p* < 0.05). One region of interest emerges at −12, −82, 26 (scale is from 1.5 to 4) in the left V2 visual cortical area. (Right) Scatter plot showing the significant relationship between GABA and visual plasticity response (*r* = 0.622, *p* = 0.013) in the left V2 region of interest in SZ only (▲) compared to HC (▲).

Between group differences in visual plasticity were observed in left V2 with region of interest at −12, −82, 26 (*p* = 0.003 FWE-corrected, *T* = 3.72) such that healthy controls had greater visual plasticity compared to SZ (Figure 3).

### Regression Analyses

As previously described by Wijtenburg et al. (11), higher glutamine was related to greater visual plasticity in healthy controls. In SZ, there were no significant regions of interest that demonstrated visual plasticity therefore the relationships between glutamine, glutamate, or GABA with visual plasticity were not assessed. However, the left V2 region of interest from the group difference analysis was significantly correlated with GABA in the SZ group (*r* = 0.622, *p* = 0.013) with significance set to *p* = 0.05/3 for the 3 metabolites of interest (Table 3). Higher levels of GABA were associated with greater visual plasticity response in the left V2 region of interest in SZ.

**Table 3:** Correlations between left V2 region of interest (ROI) and metabolites in SZ.

	***r***	***p*-value**
Left V2 ROI and Glutamate	0.18	0.51
Left V2 ROI and Glutamine	0.23	0.42
Left V2 ROI and GABA	0.62	0.013^*^

* *p < 0.05/3*.

### Correlations With Cognitive Function and Symptom Ratings

Since visual plasticity was not observed in any regions of interest in adults with SZ, there were no relationships to be explored between cognitive function and symptom ratings. Using the left V2 region of interest from the group difference analysis, there were no significant relationships between the left V2 region of interest and cognitive function with *p* = 0.05/3 (for three cognitive function variables). Further, there was a relationship between visual plasticity in the left V2 region of interest and Brief Negative Symptom Scale score (*r* = −0.510, *p* = 0.044) that did not survive correction for multiple comparisons.

## Discussion

This study reports for the first time impaired visual plasticity, assessed with fMRI, in adults with SZ compared to healthy controls. While no significant visual plasticity regions of interest survived FWE correction in SZ, group difference analyses revealed a significant visual plasticity region of interest in the left V2. This visual plasticity response region of interest was positively related to GABA levels in SZ. As a whole, these add to the body of evidence implicating altered LTP-like phenomena in SZ.

The study results add to the growing body of literature surrounding impaired visual plasticity in SZ. The majority of EEG studies examining visual plasticity in SZ and healthy controls utilized a flashing checkerboard during the high frequency stimulation component of the protocol (8, 13, 14). Similarly, the two studies that showed visual plasticity in healthy controls also used the same paradigm as part of a fMRI task (9, 11). In contrast, a recent paper by Wynn et al. reported comparable visual plasticity in both healthy control and SZ groups using EEG (16). There were two major differences between the Wynn et al. study and this study that may account for the different study findings: the high frequency stimulus and the time when low frequency stimulation was sampled post-high frequency stimulation. Wynn et al. used a set of vertical or horizontal gratings and recorded post-high frequency stimulation visual evoked potentials 30 min after the high frequency stimulation. Thus, the differences in high frequency stimulus and sampling intervals could account for the differing results.

One question is whether there was a blunted or complete lack of visual plasticity in the SZ group compared to healthy controls. From the double subtraction analysis, examination of the subject level data in the SZ group revealed that 12 of the 17 subjects did show visual plasticity in visual cortical area V3; however, the finding was significant at *p* < 0.01 uncorrected. Upon further inspection, there was a difference between participants that had a visual plasticity response and participants that did not have a visual plasticity response on the negative symptom subscale of the Brief Psychiatric Rating Scale (*p* = 0.042) such that participants with a visual plasticity response had greater negative symptom severity than those that did not have a plasticity response. There were no other differences between participants that had a visual plasticity response and participants that did not have a visual plasticity on measures of cognitive function, symptom ratings, or chlorpromazine equivalents (all *p*'s > 0.05). The varied amount of visual plasticity in the patient group reflects the heterogeneity of the illness.

While there were no relationships between the glutamatergic metabolites with visual plasticity in SZ, there were significantly higher levels of glutamate and glutamine in SZ compared to healthy controls in the occipital cortex. Three previous studies in the occipital cortex region over a range of illness durations have not found any glutamate to creatine ratio or glutamate+glutamine to creatine ratio differences (34–36). Our data are the first to show an elevation in these metabolites in the SZ group compared to the healthy control group. This may be due to the fact that our sequence is specifically optimized for glutamatergic metabolite detection; whereas the three previous studies used a spectral editing technique that cannot separate glutamate from glutamine. In terms of GABA in the occipital cortex region, previous study findings are mixed when comparing healthy controls and SZ in that lower GABA to creatine ratio in a SZ group was found in mixed illness duration SZ group (34) to no differences in first episode SZ (35) or chronic group when removing those on anticonvulsants (36). This study also found no significant GABA differences between healthy controls and SZ. Our previous work in healthy controls suggested that optimal basal levels of Glu and Gln are necessary for plasticity and in particular, higher Gln was related to greater visual plasticity response (11). Despite higher levels of glutamate and glutamine, there was no significant visual plasticity in SZ that survived FWE-correction. However, there was one region of interest that was significant at *p* < 0.01 uncorrected, but there were no significant correlations between the visual plasticity region of interest in SZ and any of the three metabolites (*p*'s > 0.5). Further, there were no significant differences in glutamate, glutamine, or GABA levels between adults with SZ that had visual plasticity and those that did not.

The mechanism underlying the relationship between higher levels of GABA associated with higher visual plasticity in the left V2 in SZ implies that inhibition plays a role in plasticity mechanisms consistent with non-human animal work (37–39). In a previous study using less stringent multiple correction criteria for the region of interest analyses, a similar positive relationship between visual plasticity and GABA levels was observed in healthy controls (11). Here, a similar relationship observed in SZ suggests GABAergic inhibition may influence excitation necessary for visual plasticity and lower GABA levels consistent with alterations in the GABAergic system are documented in the SZ literature (40–42). Examining a rough estimate of excitation/inhibition balance in the occipital cortex (GABA/Glu and GABA/Gln) revealed a trend level difference in GABA/Gln such that GABA/Gln was lower in SZ compared to healthy controls (see Supplementary Material). Further, both ratios were related at trend level (see Supplementary Material) to higher visual plasticity response in SZ only and not in healthy controls. Given the highly complex nature of excitation/inhibition balance (43, 44), these exploratory findings plus the main findings of the manuscript must be interpreted with caution given the small sample size. More studies are needed to thoroughly investigate the inhibition/excitation as it relates to visual plasticity in SZ.

There are several limitations to this study. Glutamate and GABA levels measured *via* magnetic resonance spectroscopy are a reflection of multiple pools involved in neurotransmission and other mechanisms (e.g., protein synthesis, glutathione formation, etc.). All but one of our SZ participants were taking antipsychotic medications at the time of the study, and the exact effects of these medications on visual plasticity remains unknown. A limitation of the study was that visual attention was not assessed during the fMRI portion of scan. While participants were reminded before each run to stare at the crosshair in the middle of the screen, future studies may benefit from incorporating a means of ensuring attention such as a button to a specific stimulus similar to (13, 16). Another limitation of the study was that *post-hoc* power calculations revealed that the significant findings were underpowered (61–75%) compared to the standard of 80%. Significant results in this study should be interpreted with caution as further studies are needed with a larger sample size to definitively answer whether visual plasticity is different in SZ and whether baseline metabolite levels in the occipital cortex are related to visual plasticity in SZ. A final limitation is that the adults with SZ are in the chronic phase of the illness, and these study findings may not translate to first episode SZ or those at clinical high risk for psychosis. Future studies are needed to assess visual plasticity response across the illness course.

Thus, this fMRI study tested a visual stimulation paradigm in SZ, and the results further supports several previous EEG studies that show adults with SZ have reduced response to high frequency visual stimulation. The results also show that unlike in healthy controls, glutamatergic levels did not predict plasticity activations in SZ. Future studies may utilize pharmacological or brain stimulation (e.g., TMS) interventions that modulate the glutamatergic or GABAergic systems and potentially improve visual plasticity response.

## Data Availability Statement

The datasets generated in this article are not readily available because anonymized data sharing was not included in the consent form for the study. Requests to access the datasets should be directed to S. Andrea Wijtenburg, awijtenburg@som.umaryland.edu.

## Ethics Statement

The studies involving human participants were reviewed and approved by University of Maryland Institutional Review Board. The patients/participants provided their written informed consent to participate in this study.

## Author Contributions

LR designed the study. SK collected the data. FK, JW, FG, and HC analyzed the data. LR and SW interpreted the results. SW wrote the manuscript. All authors contributed to the article and approved the submitted version.

## Conflict of Interest

The authors declare that the research was conducted in the absence of any commercial or financial relationships that could be construed as a potential conflict of interest.
