# Supplementary material for: Multimodal Neuroimaging Study of Visual Plasticity in Schizophrenia
Source: Front Psychiatry. 2021 Apr 1;12:644271. doi: 10.3389/fpsyt.2021.644271 (PMC8046908; doi:10.3389/fpsyt.2021.644271)
Supplement: Supplementary file 1 [file Data_Sheet_1.docx]

Supplement

Exploratory analyses were conducted to perform a rough estimate of excitatory/inhibitory balance in the sample with the caveat that Glu and GABA measured with magnetic resonance spectroscopy are made up of multiple pools, not exclusively neurotransmission. Group difference analyses (t-test) were performed for GABA/Glu and GABA/Gln and correlation analyses were computed for GABA/Glu and GABA/Gln with the left V2 ROI. Given that these analyses are exploratory, significance was set to p < 0.05. There were no significant differences between adults with SZ and healthy controls for GABA/Glu (p=0.176), but there was a trend level difference between groups for GABA/Gln (p=0.052) such that adults with SZ had lower GABA/Gln than healthy controls. Correlation analyses between the left V2 ROI with GABA/Glu and GABA/Gln were trend level significant at r=0.467, p=0.079 and r=0.505, p=0.055 in the SZ group only


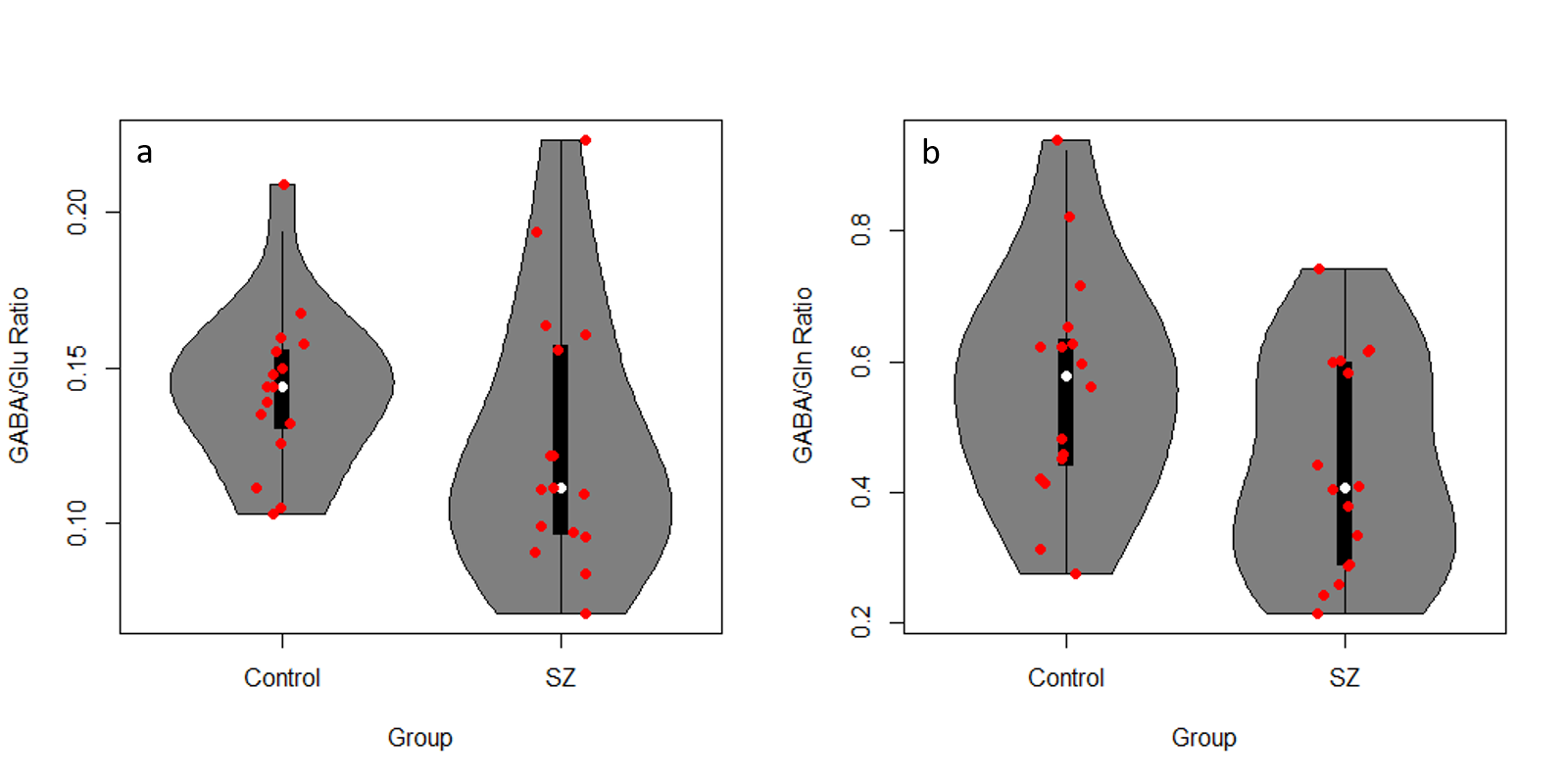


Figure S1. violin plots with individual data plots overlayed in red for (a) GABA/Glu ratio and (b) GABA/Gln ratio in adults with SZ and healthy controls.
